# Ovarian aging: pathophysiology and recent developments in maintaining ovarian reserve

**DOI:** 10.3389/fendo.2025.1619516

**Published:** 2025-10-02

**Authors:** Mana Hirano, Takako Onodera, Kazuki Takasaki, Yuko Takahashi, Takayuki Ichinose, Haruka Nishida, Haruko Hiraike, Kazunori Nagasaka

**Affiliations:** Department of Obstetrics and Gynecology, Teikyo University School of Medicine, Tokyo, Japan

**Keywords:** ovarian aging, ovarian reserve, fertility preservation, follicle depletion, hormonal therapies, menopause, delayed parenthood, biomarker-driven

## Abstract

According to the World Health Organization, infertility has emerged as a critical public health issue, affecting approximately 48 million couples and 186 million individuals worldwide. Ovarian aging—defined by the progressive depletion and functional deterioration of the primordial follicle pool—accounts for a major proportion of female−factor infertility and has profound socioeconomic consequences. It is characterized by a decline in follicle quantity and quality, which significantly influences infertility. This phenomenon is multifaceted, involving genetic predisposition, hormonal fluctuations, mitochondrial dysfunction, oxidative stress, and ovarian microenvironment alterations. This review explores the biological mechanisms of ovarian aging, evaluates current therapeutic advances, and identifies strategies to maintain ovarian function and prolong reproductive lifespan. Recent advancements—including antioxidant and mitochondria-targeted therapies, hormonal modulation, growth factor interventions (e.g., platelet-rich plasma), mitochondrial transfer, and *in vitro* follicle activation—show promise for maintaining ovarian reserve. Fertility preservation strategies, such as ovarian tissue cryopreservation and transplantation, and pharmacological inhibition of follicle depletion, have expanded therapeutic options. The development of personalized treatments, refined biomarkers, and integrative strategies combining antioxidants, hormonal therapies, and novel fertility preservation techniques is essential. Therefore, translational research utilizing animal models remains crucial for validating efficacy and safety prior to clinical application. Future research should prioritize validating these emerging therapies through larger clinical trials to ensure safe, effective, and practical translation into clinical practice, ultimately prolonging reproductive lifespan and enhancing quality of life for aging women.

## 1 Introduction

**Figure 1:**
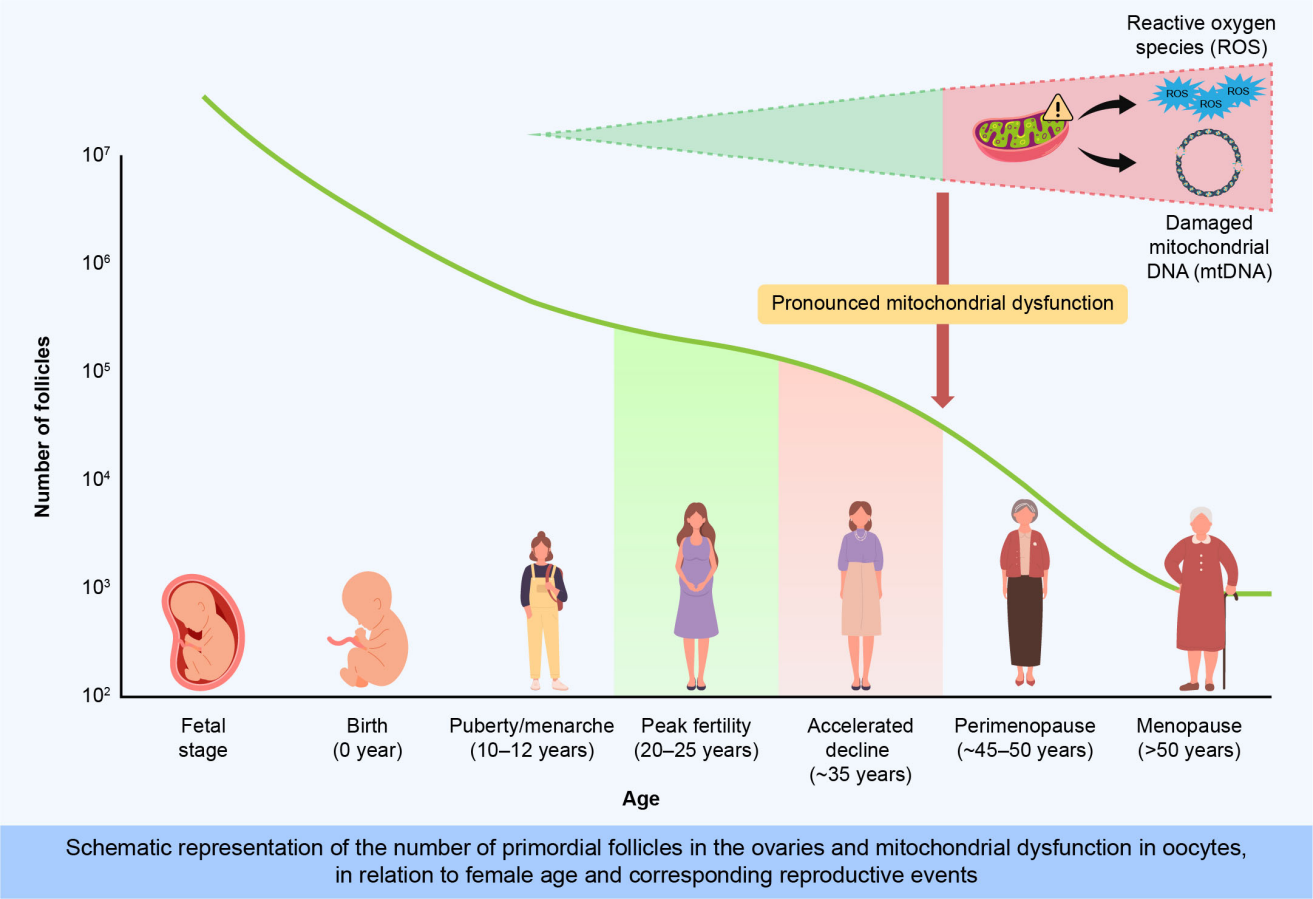
Age-dependent decline in primordial follicle number and mitochondrial dysfunction associated with ovarian aging.This illustration depicts the progressive depletion of primordial follicles from fetal development through menopause. At birth, approximately 1–2 million primordial follicles remain, declining sharply to about 300,000 by menarche and rapidly decreasing after the mid-30s. Concurrently, oxidative stress (ROS), mitochondrial DNA (mtDNA) damage, and genetic factors accelerate ovarian aging, impairing oocyte quality and reproductive capacity.

The age of marriage and childbearing is rising globally with the increasing participation of women in the workforce, contributing significantly to higher infertility rates. Additionally, the average age of women experiencing their first pregnancy and childbirth has steadily increased over the past decades (1). According to the World Health Organization, infertility affects approximately 48 million couples and 186 million individuals worldwide, posing a critical public health issue. The economic implications of infertility treatment place substantial financial burdens on both individuals and healthcare systems, highlighting the urgent need for effective management and preventive strategies. Delayed parenthood also impacts family dynamics and increases psychosocial stress for individuals facing infertility, further emphasizing the importance of addressing ovarian aging. Ovarian aging, which is characterized by declining follicle quantity and quality, significantly contributes to infertility. At approximately 20 weeks of gestation, women possess approximately 7 million follicles, which decline to 1–2 million at birth and further decrease to 300,000–400,000 by the time of menarche. Menopause typically occurs when approximately 1,000 follicles remain (2). After the mid-30s, the rate of follicle depletion accelerates, increasing the risks of aneuploidy and adverse pregnancy outcomes. Follicular development, regulated by gonadotropin-releasing hormone (GnRH), spans from primordial to ovulatory follicles, with a typical duration of approximately 150 days or more; however, most follicles undergo atresia before ovulation (3). Ovulation occurs approximately 400 times from the time of menarche to menopause. Menopause usually occurs at ages 51–52 years and varies across ethnicities; it is preceded by a transitional period known as perimenopause, lasting 4–8 years with irregular cycles and intensified hormonal fluctuations (4). The hypoestrogenic state of menopause triggers vasomotor symptoms and increases osteoporosis and cardiovascular risks (**Figure 1**).Primordial follicles, which constitute the initial follicle pool, remain quiescent until activated for growth, a process crucially regulated by intricate cellular and molecular signaling networks. Recent advances have elucidated that primordial follicle activation is primarily initiated by somatic primordial follicle granulosa cells (pfGCs). This activation involves critical signaling pathways, notably the mechanistic target of rapamycin complex 1 (mTORC1) within pfGCs, promoting their differentiation and proliferation. Activation of mTORC1 in pfGCs subsequently enhances the secretion of KIT ligand (KITL), which binds to KIT receptors on dormant oocytes, triggering intra-oocyte phosphatidylinositol 3 kinase (PI3K) signaling essential for oocyte awakening and follicular growth (5). Understanding these precise regulatory mechanisms underlying ovarian aging is critical to developing interventions aimed at preserving ovarian reserve and mitigating associated socioeconomic consequences (6, 7). Therefore, understanding the mechanisms underlying ovarian aging is critical to developing interventions aimed at preserving ovarian reserve and mitigating associated socioeconomic consequences. This review explores the biological mechanisms of ovarian aging, evaluates current therapeutic advances, and identifies strategies for maintaining ovarian function and prolonging reproductive lifespan.

## 2 Pathophysiology of ovarian aging

### 2.1 Follicular depletion

Humans are born with a finite pool of approximately 600,000 primordial follicles that diminish progressively from fetal life through menopause (6). These follicles are continually activated and recruited for follicular development until menopause (7). At menarche, approximately 800–900 primordial follicles are recruited each month, decreasing to <100 by menopause (8). While atresia (apoptosis) is the predominant mechanism of follicle loss, external factors, such as oxidative stress and environmental toxins, have been shown to accelerate depletion. Lifestyle factors, including smoking, alcohol consumption, poor nutrition, and exposure to environmental chemicals, further expedite follicular depletion, potentially causing early menopause and decreased fertility (9). Consequently, understanding these dynamics is crucial for developing interventions aimed at preserving ovarian function and fertility.

### 2.2 Hormonal dysregulation

A decline in follicle numbers leads to reduced ovarian hormone production, notably inhibin B and anti-Müllerian hormone (AMH). This reduction diminishes negative feedback on follicle-stimulating hormone (FSH), resulting in elevated serum FSH levels. Additionally, persistent gonadotropin imbalances exacerbate follicular depletion, creating a detrimental cycle (7, 8).

### 2.3 Mitochondrial dysfunction and oxidative stress

Oocytes heavily depend on mitochondrial function for adenosine triphosphate (ATP) generation. Age-related mitochondrial DNA (mtDNA) damage, including mutations, oxidized bases, and copy number abnormalities, compromises oocyte competence and fertility potential (10–14). Primary oocyte division halts at prophase I during fetal life and resumes at menarche, which is a prolonged resting period that predisposes mitochondria to mutations (10). Reportedly, patients exhibiting mitochondrial dysfunction typically have lower AMH levels and follicular numbers than healthy individuals (12–14).

### 2.4 Reactive oxygen species

Reactive oxygen species (ROS), which are byproducts of normal mitochondrial metabolism, induce DNA damage, accelerate follicular attrition, and contribute to age-related fertility decline. Excess oxidative stress leads to follicle atresia and diminished oocyte quality (15). Older patients with infertility exhibit lower superoxide dismutase levels in granulosa cells, highlighting decreased antioxidant efficacy with ovarian aging (16). They also exhibit 35% lower granulosa−cell superoxide−dismutase activity than age−matched fertile controls (15, 17). Lifestyle factors, including smoking and alcohol, additively increase ROS production, while antioxidants, such as vitamins C and E and dimethyl fumarate (DMF), potentially mitigate oxidative stress (17, 18). Sirtuins, particularly sirtuin 1 and sirtuin 3, also protect ovarian function by enhancing antioxidant defenses and DNA repair processes (19–21).

### 2.5 Genetic and epigenetic factors

Genetic mutations associated with premature ovarian insufficiency (POI) include those in the FSH receptor, steroidogenic acute regulatory protein, and forkhead transcription factor 2 (FOXL2), alongside chromosomal abnormalities. FOXL2 mutations have been shown to impair granulosa cell differentiation and follicular growth, significantly reducing follicle viability (8). Epigenetic changes, including DNA methylation alterations and histone modifications, further disrupt essential gene regulation pathways critical for folliculogenesis and oocyte quality (8, 22).

### 2.6 Microenvironmental alterations

Microenvironmental alterations changes in the ovarian microenvironment, such as increased fibrosis, reduced angiogenesis, chronic inflammation, and immune cell infiltration, significantly impact follicle survival. These alterations, which are influenced by oxidative stress, hormonal fluctuations, aging, environmental toxins, and repeated ovulatory cycles, impair the delivery of nutrients and growth factors required for follicular development, thereby accelerating ovarian aging and follicular loss (23). Single−cell RNA−sequencing has revealed an age−dependent shift toward pro−fibrotic macrophage subsets within the ovarian stroma (23).

## 3 Recent developments in maintaining ovarian reserve

### 3.1 Pharmacological inhibition of follicle recruitment

While GnRH agonists and antagonists are employed to mitigate chemotherapy-induced ovarian damage, their effectiveness remains controversial. GnRH agonists/antagonists temporarily suppress gonadotropin secretion, potentially slowing follicular depletion. Their clinical use during chemotherapy remains controversial, with existing data presenting mixed outcomes. Recent systematic reviews and meta-analyses (24) emphasize the need for further clinical trials focusing on dosage optimization, timing of administration relative to chemotherapy, and patient selection criteria to definitively clarify the efficacy and clinical utility of GnRH analogs. Ongoing and future clinical studies could significantly advance our understanding, potentially transforming fertility preservation strategies (23–25). However, the protective value of GnRH analogs during chemotherapy remains debated, and the latest network meta−analysis identifies significant heterogeneity across cancer types (25, 26).

### 3.2 Hormonal and growth factor modulation

Dehydroepiandrosterone (DHEA) supplementation has demonstrated improved ovarian reserve markers such as serum AMH, inhibin B, and antral follicle count (AFC), consequently enhancing *in vitro* fertilization outcomes for patients with diminished ovarian reserves. However, individual response variability necessitates further studies to refine dosing regimens, duration, and safety profiles (27). Recent studies between 2023 and 2025 have also increasingly highlighted the roles of insulin-like growth factor (IGF) and vascular endothelial growth factor (VEGF) in modulating follicular development and responsiveness during assisted reproductive technologies. Emerging clinical trials and in-depth mechanistic analyses underline the promising therapeutic potential of these growth factors, warranting extensive clinical validation to further refine their clinical application (28). Notably, supplementation with DHEA, IGF−I, and VEGF each improved AFC by 12–25% in small randomized clinical trials (RCTs) (26, 27). Combination−based therapy—tailoring growth−factor cocktails to individual biomarker profiles—is emerging as a next step.

### 3.3 Antioxidant and mitochondrial therapies

Numerous clinical studies have shown improved pregnancy outcomes and reduced oxidative stress through antioxidant therapies, such as Coenzyme Q10, resveratrol, melatonin, and DMF, which enhance mitochondrial function, reduce ROS and improve oocyte quality. Melatonin specifically delays post-ovulatory aging by upregulating the expression of antioxidant enzymes (29, 30). A rodent RCT showed that mitoquinone reduced ROS and preserved follicular density after ovarian−tissue vitrification, supporting its adjunctive use during ovarian tissue cryopreservation (OTC) (31). Elamipretide, a cardiolipin−binding peptide, is in phase III trials for mitochondrial disorders and has entered preclinical reproductive studies; its proposed mechanisms include restoration of ATP synthesis and reduction of lipid peroxidation (32). Recent literature extensively explores advanced mitochondrial-targeted antioxidants, including mitoquinone and elamipretide, highlighting their effectiveness in protecting mtDNA integrity, maintaining mitochondrial function, and enhancing energy metabolism within oocytes, consequently prolonging reproductive lifespan and improving clinical outcomes (31, 32). While experimental mitochondrial transfer techniques also show substantial potential, efficiently replacing mutant oocyte mtDNA and significantly enhancing embryo viability, clinical validation and safety assessments remain necessary (11).

### 3.4 *In vitro* activation of primordial follicles

Platelet-rich plasma (PRP) injections have emerged as a promising approach to activate dormant follicles and clinically improve fertility outcomes, particularly in patients with diminished ovarian reserves. A 2025 systematic review of 542 women with POI or poor ovarian response demonstrated that intra−ovarian PRP injections raised AMH by a pooled mean ± standard deviation of 0.32 ± 0.10 ng mL^-^¹ and yielded a 9% clinical−pregnancy rate (33). Similarly, *in vitro* activation (IVA), which modulates the phosphatase and tensin homolog/phosphatidylinositol 3−kinase/protein kinase B signaling pathway, has successfully induced follicle activation in patients with POI. However, inconsistent clinical outcomes underscore the necessity for protocol standardization, biomarker identification, and downstream follicle development technique optimization. Advances in these areas could significantly enhance the clinical efficacy and applicability of IVA, marking a significant leap forward in fertility preservation strategies (34–36).

### 3.5 Forkhead transcription factor 2 and estrogen receptor beta–progesterone receptor

Axis interactions between FOXL2, estrogen receptor beta (ERβ), and progesterone receptor (PR) critically regulate follicular dynamics, granulosa cell function, and ovarian identity. However, mutations or altered expression of FOXL2 severely disrupt follicular viability and can lead to granulosa cell tumors. Selective PR modulators, such as ulipristal acetate (UPA), demonstrate dual effects—enhancing follicular growth yet inhibiting ovulation by suppressing luteinizing hormone surge. Therefore, ongoing studies are necessary to comprehensively evaluate the long-term impact of UPA on ovarian reserve, considering its potential therapeutic benefits and safety concerns. Advanced genomic and proteomic analyses continue to elucidate these complex interactions, potentially facilitating the development of targeted therapies that can safely and effectively modulate these pathways to preserve ovarian function (37–39). New cryogenic chromatin immunoprecipitation studies have mapped >1,500 FOXL2−ERβ co−occupied enhancers that govern granulosa−cell identity (22). Moreover, selective PR modulators, such as UPA, hold promise but require ovulation−safety profiling (39).

### 3.6 Ovarian tissue cryopreservation and transplantation

OTC and transplantation represent promising fertility preservation strategies, effectively restoring ovarian endocrine function and enabling successful pregnancies following gonadotoxic treatments such as chemotherapy and radiotherapy. OTC involves surgically removing ovarian tissue prior to treatment, freezing the tissue, and subsequently transplanting the thawed tissue back into the patient after therapy completion. Recent clinical advancements have shown improved graft survival rates, enhanced follicular viability, and successful long-term endocrine restoration and fertility outcomes (40). Nonetheless, OTC still poses significant ethical and regulatory challenges, including the risks of malignancy recurrence due to potential re-implantation of malignant cells, consent complexities, equitable access, and clinical standardization. Current and future research priorities should address these challenges by developing enhanced grafting techniques to reduce ischemic injury, improving tissue preservation protocols to enhance follicular viability, rigorous pre-transplant screening methods for residual malignancy, and comprehensive long-term follow-up to ensure patient safety. Furthermore, establishing clear and stringent patient selection criteria, standardized informed consent processes, and equitable access frameworks is critical for the broader and safer clinical implementation of OTC. Continued multidisciplinary research efforts are essential to refine OTC protocols and ultimately expand the accessibility and effectiveness of fertility preservation options for patients receiving gonadotoxic treatments (41). The seminal pronuclear−transfer study by Kang et al (11). has been followed by a United Kingdom cohort in which 22 women underwent pronuclear transfer, resulting in eight healthy live births with negligible heteroplasmy (42). However, long−term registries remain imperative to monitor heteroplasmy drift.

## 4 Challenges and future perspectives

**Figure 2:**
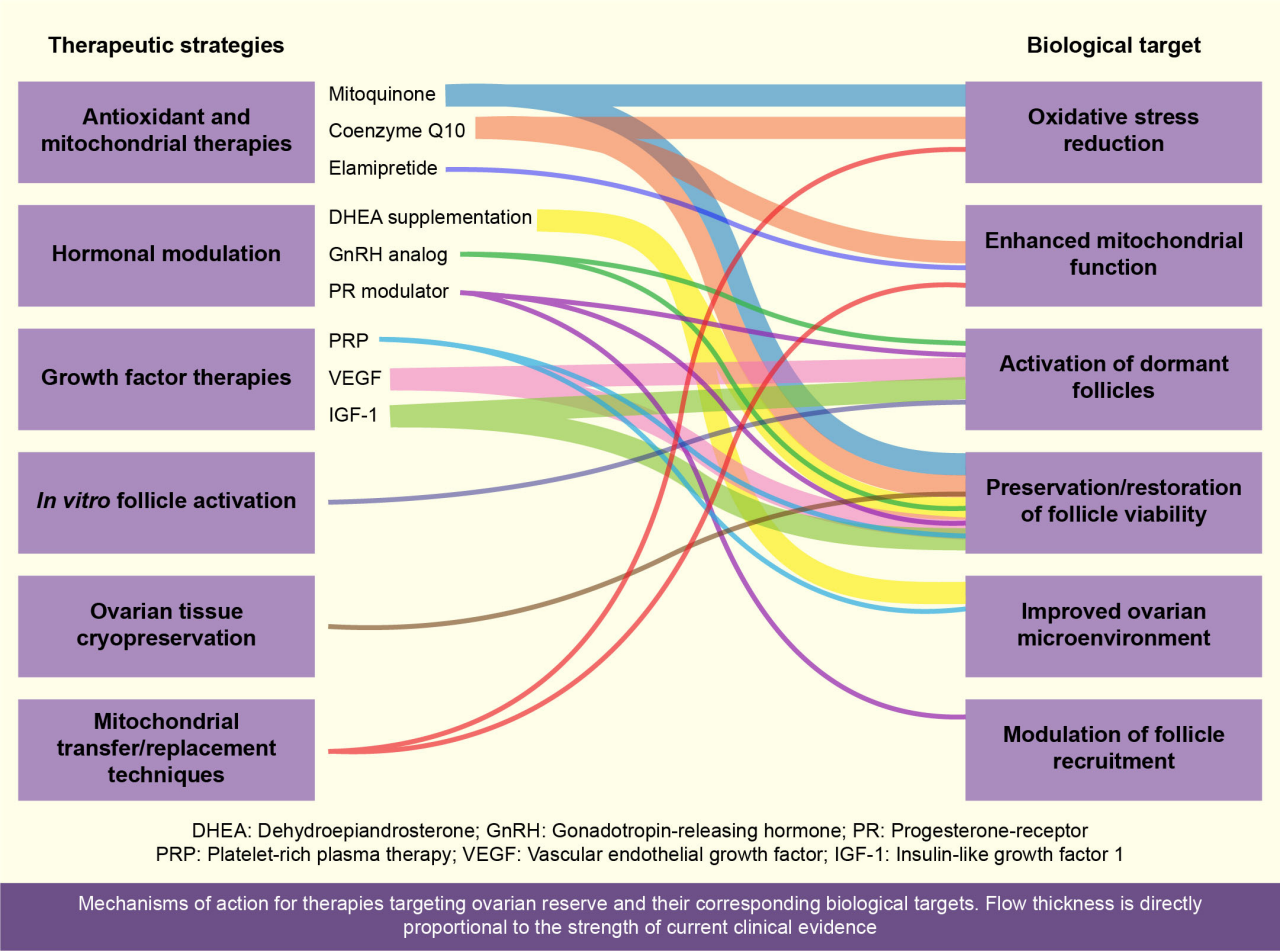
Overview of current therapeutic strategies aimed at preserving or restoring ovarian reserve (**Figure 2**). This schematic illustrates various therapeutic strategies for maintaining ovarian reserve, highlighting antioxidant therapies (Coenzyme Q10, resveratrol, melatonin, dimethyl fumarate [DMF]), mitochondrial transfer techniques, hormonal modulation (DHEA, platelet-rich plasma [PRP]), pharmacological agents (UPA, GnRH analogs), *in vitro* activation (IVA) via the PTEN-PI3K-AKT pathway, and ovarian tissue cryopreservation. These approaches aim to enhance oocyte quality, promote follicular activation, and preserve fertility, particularly after cancer treatment or delayed motherhood.

Recent developments in maintaining ovarian reserve encompass numerous challenges that primarily revolve around efficacy, clinical validation, safety, and ethical considerations, any of which are closely related to the therapeutic strategies summarized in **Figure 2**. The following subsections address key issues and future research directions:

### 4.1 Ethical considerations

Ethical concerns related to fertility preservation methods, including OTC, mitochondrial transfer, and genome editing technologies, pose significant challenges. Potential risks of malignancy recurrence, particularly with ovarian tissue transplantation, require careful ethical evaluation. Equitable access to fertility preservation, particularly for younger patients, also remains a crucial ethical challenge. Emerging ethical debates focus on reproductive autonomy, informed consent complexities, and resource allocation. Furthermore, recent ethical frameworks and guidelines propose clearer protocols for informed consent, particularly addressing nuances of potential long-term health implications and intergenerational ethical concerns related to genome editing (43).

### 4.2 Endocrine modulation strategies

Optimizing endocrine modulation therapies, including hormonal supplements (DHEA) and growth factors (IGF and VEGF), requires further refinement and clinical validation. Current research emphasizes individualized treatment strategies to account for significant patient variability. Notably, recent advances highlight the potential of novel endocrine modulators to optimize follicular responsiveness and improve outcomes in assisted reproductive technologies. However, future clinical trials should focus on identifying reliable biomarkers to predict patient responsiveness, refining dosing regimens, and ensuring long-term safety. Ongoing research in endocrine modulation continues to provide promising insights, warranting comprehensive exploration in future clinical settings (44, 45).

### 4.3 International regulatory frameworks

International regulatory challenges significantly impact the clinical implementation of novel ovarian preservation techniques. Additionally, variability in regulatory standards among countries presents barriers to harmonized clinical practices. Recent discussions have underscored the necessity of establishing unified regulatory guidelines to facilitate global adoption and ensure patient safety across different jurisdictions. Proposed frameworks suggest rigorous regulatory oversight for emerging reproductive technologies, including mitochondrial transfer and genome editing, to ensure safety, ethical integrity, and clinical effectiveness (46). Collaborative efforts across regulatory bodies, such as the Food and Drug Administration (FDA), European Medicines Agency(EMA), and Pharmaceuticals and Medical Devices Agency (PMDA), are essential to develop consensus guidelines addressing patient safety, equitable access, and standardized clinical protocols.

Future research directions should prioritize robust clinical trials addressing these challenges, specifically focusing on long-term safety, efficacy validation, ethical considerations, and standardized regulatory frameworks. Furthermore, multidisciplinary research strategies integrating clinical, ethical, regulatory, and patient-centered perspectives are vital to translate innovative fertility preservation strategies into routine clinical practice.

## 5 Conclusion

Ovarian aging is a multifaceted biological phenomenon influenced by genetic predisposition, hormonal changes, mitochondrial dysfunction, oxidative stress, and alterations in the ovarian microenvironment. Recent advancements, including antioxidant and mitochondria-targeted therapies, hormonal modulation, growth factor interventions such as PRP, and innovative techniques including mitochondrial transfer and IVA, show promise for preserving ovarian reserve. Fertility preservation strategies (such as OTC) and pharmacological approaches to inhibit follicle depletion have expanded the scope of therapeutic interventions. Therefore, the development of personalized treatments, refined biomarkers, and integrative therapeutic strategies that combine antioxidants, hormonal therapies, and novel fertility preservation techniques is crucial. Translational research using animal models is indispensable for validating efficacy and safety before clinical application. Ongoing exploration and refinement in these areas strive to prolong the reproductive lifespan and improve the overall quality of life for aging women. Consequently, ovarian aging is no longer an unmodifiable fate. A convergence of antioxidant, endocrine, growth factor, and cellular therapies—implemented within clear regulatory guardrails—offers realistic prospects for prolonging female reproductive lifespan. Moreover, interdisciplinary collaboration and international policy harmonization will be pivotal to translate these advances from experimental promise to routine care.
